# Data on farmers’ adoption of climate change mitigation measures, individual characteristics, risk attitudes and social influences in a region of Switzerland

**DOI:** 10.1016/j.dib.2020.105410

**Published:** 2020-03-10

**Authors:** Cordelia Sophie Kreft, Robert Huber, David Johannes Wüpper, Robert Finger

**Affiliations:** Agricultural Economics and Policy Group, ETH Zurich, Zurich, Switzerland

**Keywords:** Agricultural climate change mitigation, Farmers’ climate change attitudes and perceptions, Risk attitudes, Non-cognitive skills, Social networks, Switzerland

## Abstract

We present survey data on the adoption of agricultural climate change mitigation measures collected among 105 farmers in a region in Switzerland in 2019. We surveyed measures farmers use to reduce greenhouse gas emissions on the farm level. The list comprised 13 measures related to energy production and use, herd and manure management as well as crop production. Additionally, data was collected with regard to farmers’ individual concerns and perceptions of climate change, attitudes and goals, self-efficacy and locus of control, income satisfaction and social influences. Moreover, risk preferences as well as loss aversion and probability weighting were elicited using a multiple price list. The survey data was matched with cantonal farm census data, containing information on farm size, farm type and age of the farmers.

Specifications table

**Table:** 

Subject	Agricultural economics
Specific subject area	Adoption of agricultural climate change mitigation measures, non-cognitive skills, social influences, risk preferences
Type of data	CSV file
How data were acquired	Online survey combined with farm census data Limesurvey R
Data format	Raw and partly filtered (for reasons of confidentiality)
Parameters for data collection	The survey targeted farmers of all production types in a specific region of Canton Zurich, Switzerland
Description of data collection	The online questionnaire was distributed via Limesurvey to 389 farmers registered in the region of Zürcher Weinland, Canton Zurich, Switzerland. Risk preferences, loss aversion and probability weighting were elicited using a multiple price list. Participation was incentivized. In total, 105 respondents completed the survey. The data was anonymized.
Data source location	Zürcher Weinland, Canton Zurich, Switzerland
Data accessibility	Data is accessible via ETH Zürich Research Collection: http://hdl.handle.net/20.500.11850/383116

## Value of the data

• The data highlights which measures are taken by farmers to reduce greenhouse gas emissions and elicits climate change related perceptions, attitudes, self-efficacy, locus of control, social influences as well as risk preferences. Risk preferences, loss aversion and probability weighting were elicited by a multiple price list.
• The data enables to understand the adoption of climate change mitigation measures in agriculture and associate it to farmers’ individual characteristics as well as farm structural characteristics.
• The data can be used to analyse drivers of agricultural mitigation measures including a wide range of behavioural factors and farm characteristics. This allows for a broad range of control variables.
• The data allows to interlink different individual factors, e.g., non-cognitive skills and risk preferences or climate change perceptions and concerns.

## 1 Data description

We collected survey data on climate change mitigation measures adopted by farmers and combined them with cantonal farm census data. Mitigation measures in the survey were selected based on relevant literature and suitability for Swiss agriculture [1,2]. The here presented data is based on a combination of census and survey data, which were matched by farmers’ email addresses.^1^ It contains information on the adoption of greenhouse gas reduction measures, on farm structure and production as well as individual farmers’ characteristics. For reasons of confidentiality, any personally identifiable information (i.e., all qualitative data, names and contact details of respondents, names of other persons as well as personal feedback) was removed from the dataset. Risk preferences, loss aversion and probability weighting were elicited using a multiple price list following the approach of Tanaka et al. [3]. The original questionnaire, the dataset and the codebook describing the variables are available on the ETH Zürich Research Collection: http://hdl.handle.net/20.500.11850/383116.

## 2 Experimental design, materials, and methods

**Fig. 1:**
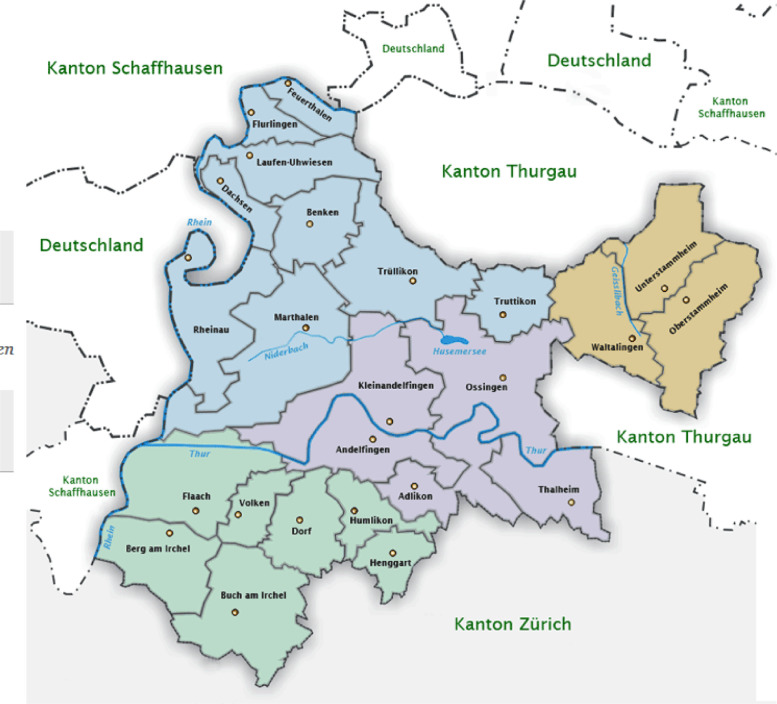
Map of the region of Zürcher Weinland including 24 municipalities (https://www.feuerthalen.ch/tourismus/umgebung/zuercher-weinland.html/323).

The online survey (in German) was distributed in March 2019 via a link sent by email to all 389 farmers in the region of Zürcher Weinland, Canton Zurich, Switzerland, that were registered with cantonal authorities. The region includes 24 municipalities and is part of the political district of Andelfingen (Fig. 1).

The email was accompanied by a supporting letter of the Cantonal Farmers Union. We used the online platform Limesurvey^2^ to design the survey and collect the data. The questionnaire was tested and improved in two rounds of pre-tests. First, we tested general wording, understanding and user-friendliness with six students of agricultural sciences. In a next step, we obtained content-related feedback from ten farmers at the farming school of the Canton of Zurich. The survey was online for eight weeks. Two reminders were sent to farmers who had not filled out the questionnaire after two and four weeks. As an incentive to participate, we provided summary information on the first survey results. Moreover, farmers were given CHF 10 for answering all questions. Additionally, farmers had the chance to win up to CHF 190 based on real payouts from the lottery (multiple price list).

The questionnaire contained 26 questions and on average, farmers needed 40 min to complete the survey. The survey was structured in following sections:

(i) Expected consequences and perceptions of climate change;
(ii) Perceived self-efficacy and locus of control (non-cognitive skills);
(iii) Current implementation and expected effectiveness of mitigation measures;
(iv) Education, personal preferences, goals and innovativeness;
(v) Income satisfaction;
(vi) Personal social networks and social comparison;
(vii) Risk preferences, loss aversion and probability weighting (multiple price list).

### 2.1 Expected consequences and perceptions of climate change

Farmers were asked if they expected negative or positive consequences of climate change with regard to the overall agricultural sector in Switzerland and the economic future of their own farm. Moreover, farmers were asked to indicate whether over the past 10 years they had experienced decreases or increases in occurrence of hail, permanent droughts, frost in autumn and spring, heavy precipitation, long rainy periods and heat waves. Here, we are primarily interested in climatic changes perceived by the farmers as we assume perception of climate change to be an important factor in decision-making regarding mitigation efforts. However, as the data relate to one specific region, real climate data could be easily matched to the survey data.

### 2.2 Perceived self-efficacy and locus of control (non-cognitive skills)

We included a question containing five items on self-efficacy (3 items) and locus of control (2 items) based on [4] and [5]. All items were related to the domain of agricultural climate change mitigation.

### 2.3 Current implementation and expected effectiveness of mitigation measures

Farmers were asked to indicate which measures they undertook to reduce GHG emissions on the farm. In total, 13 measures could be selected and respondents had the option to add additional measures they adopted.^3^ For reasons of identifiability, we did not include these additional measures in the raw data. Measures were carefully chosen regarding effectiveness, relevance and suitability for Swiss agriculture based on [1,2]. The following measures were included:

– Energy production and use
  ○ Solar panels
  ○ Biogas plant
  ○ Ecodrive mode for tractor
– Livestock and manure management
  ○ Replacement of (imported) concentrate feed by domestic legumes
  ○ Reduction of concentrate feed to max. 10% of the ration
  ○ At least 5 lactation periods per dairy cow
  ○ Double purpose cattle breed
  ○ Feed of additives to reduce methane emissions from enteric fermentation
  ○ Coverage of manure storage
  ○ Composting of manure
– Crop production
  ○ Emissions reducing fertilizer application technique (e.g., drag hoses)
  ○ Cover and catch crops in rotation
  ○ Tillage without plough

For farms where a certain measure was not eligible (e.g., livestock measures for pure crop farms), respondents could chose the option “not relevant for my farm type”. Farmers were furthermore asked to indicate how effective for climate change mitigation they rated each measure (regardless of whether they adopted the measure or not). We also included a question on the potential adoption of each non-adopted measure where farmers had to indicate whether they could imagine to adopt the measure in the future or not.

### 2.4 Education, personal preferences, goals, and innovativeness

After a question about level of education, respondents were asked to indicate which agricultural activities they could generally imagine for their farms, namely dairy cows, cattle fattening, pig fattening, poultry, crop farming, specialized culture or options outside the agricultural sector. Each activity had to be rated. We also included a question on personal values, where farmers were asked to rate six different goals concerning agricultural production, namely protection of natural resources, reduction of GHG emissions, preservation of animal and plant biodiversity, high yields, generation of high agricultural income and acknowledgement from other farmers. This was followed by a question on the level of attainment of the six goals. To collect data on farmers’ innovativeness, a question containing five items was included regarding the pioneer character of respondents.

### 2.5 Income satisfaction

This section of the questionnaire contained two questions referring to the satisfaction with the agricultural income (including direct payments) and two questions referring to satisfaction with the total household income (including employment outside the agricultural sector). We asked farmers to indicate at which yearly income level (on a scale from CHF 160,000 to CHF 40,000) they would not be satisfied anymore (note that the average total income of Swiss farms is CHF 97,000, consisting of CHF 65,000 farm income and CHF 32,000 off-farm income, see e.g., www.agrarbericht.ch). We also queried the share (in percentage) of agricultural in total household income.

### 2.6 Personal social networks and social comparison

We included a question regarding the subjective importance respondents placed on the opinion of others about their own farm and farming abilities. Another question in this section concerned the importance of social comparison expressed by needs of superiority or conformity with regard to agricultural income and climate change mitigation.

Respondents were furthermore asked to list the names (acronyms and nicknames were allowed as well) of up to ten persons in their direct social network with whom they regularly exchanged about general agricultural matters and agricultural climate change mitigation. To specify the type of relation, we further queried how the respondent was connected to each person listed, namely neighbour, colleague, friend, family member, partner, club colleague, veterinary, extension service or other. In the latter case (other), respondents were asked to specify the type of connection. Lastly, we included a question on how important the opinions, attitudes and activities of each person listed were for decision-making on the farm.

### 2.7 Risk preferences, loss aversion and probability weighting (multiple price list)

**Fig. 2:**
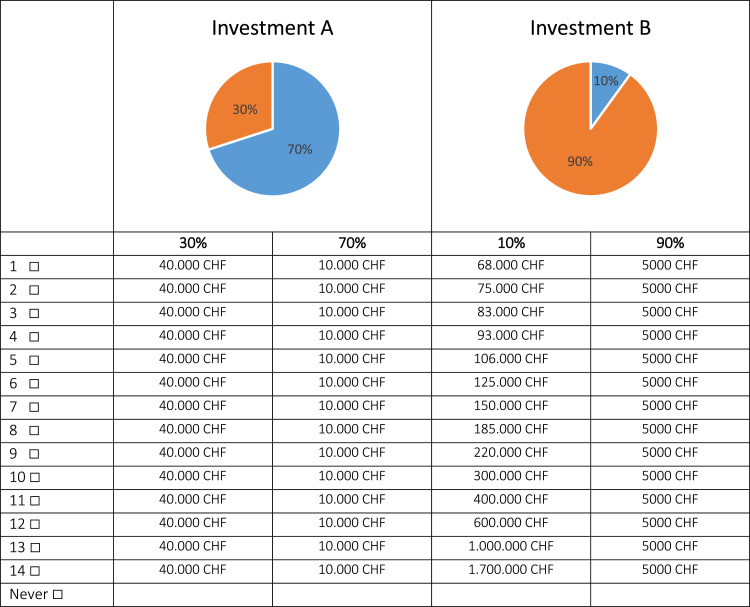
Example of multiple price list.

To elicit risk preferences, loss aversion and probability weighting, we added three multiple price lists (lottery tasks) proposed by Tanaka et al. [3] (see also [6] for an overview) (Fig. 2). The wording and level of payouts were adapted to the farming context and climate change mitigation. More precisely, farmers’ were presented the following introductory text:

“In order to implement climate change mitigation on your farm, you can decide between investing in either measure A or measure B. Both investments offer a certain return, e.g., due to higher efficiency and cost reduction. Both investments have the same price and the respective return will be paid out at the same time. In three out of ten cases (30%), investment A offers a return of CHF 40,000 and in 7 out of ten cases (70%), investment A offers a return of CHF 10,000. Investment B offers a return of CHF 68,000 in one of ten cases (10%) and in nine of ten cases (90%) a return of only 5000 CHF is offered.

The return of investment B with the lower probability (10%) is increased in the following tables. At which level of return would you be willing to take the higher risk and invest in B instead of the more stable alternative A. Note that there are no right or wrong answers – decide only according to your personal preferences.”

For ease of understanding, farmers were additionally presented a short explanatory video clip (the clip is available on the ETH research collection: http://hdl.handle.net/20.500.11850/383116).

Concerning the real payout modalities, we followed [7] and [8]. They were instructed that real payouts were based on their decisions. For every lottery, one row was randomly chosen using a macro-enabled Excel spreadsheet. Based on the decision in this randomly chosen row (investment A or B), the lottery was drawn. The final amount of all three tasks was summed up and divided by 10,000. As the third lottery could also entail a loss of up to CHF 5, participants were provided a secure endowment of CHF 5 beforehand such that they could not lose any money for real.

Participants could chose whether they wanted to receive the real gains from the lottery and give contact and bank details. All participants who chose this option were later informed about their gains via email. The theoretical minimum was CHF 3.9 (including CHF 5 security endowment from the third lottery) and the maximum win was CHF 190 (including CHF 5 security endowment). The expected return of each participant was approximately CHF 11.

## Conflict of Interest

The authors declare that they have no known competing financial interests or personal relationships that could have appeared to influence the work reported in this paper.

## Appendix Supplementary materials

Image, application 1

Image, application 2

Image, application 3

## Footnotes

1 For three of the farmers who completed the survey, there were no entries in the farm census data for the period of question (e.g., because they did not apply for direct payments in the respective period).

2 www.limesurvey.org.

3 Other measures mentioned by farmers were for example use of vegetable carbon, dilution of cattle slurry, younger age of cow at first calving, reduction of pesticides, fungus-resistant varieties, regular maintenance of machines etc.
