# Supplementary material for: Data on farmers’ adoption of climate change mitigation measures, individual characteristics, risk attitudes and social influences in a region of Switzerland
Source: Data Brief. 2020 Mar 10;30:105410. doi: 10.1016/j.dib.2020.105410 (PMC7110304; doi:10.1016/j.dib.2020.105410)
Supplement: Supplementary file 2 [file mmc2.docx]

Survey on agricultural climate change mitigation in the region of Zürcher Weinland 2019

# Introduction

Dear farmers,

Against the background of global warming, we are dealing with the options of climate protection in Swiss agriculture. In particular, we are interested in better understanding farmers’ decision-making with regard to on-farm climate change mitigation. Your assessments and personal network information, preferences and risk attitudes are central to this. Since it is about your very personal assessments, there are no wrong answers.

Completing the questionnaire takes about 30 minutes. As a reward for answering all questions, you will receive CHF 10 at the end of the survey. You can also win up to CHF 190 in the last part. If you are interested, we will gladly send you a summary of the survey results.

Your data and information will be kept strictly confidential and will be used anonymously for scientific purposes only.

We thank you very much for your participation!

For questions, please contact: Cordelia Kreft Group for Agricultural Economics and Politics ETH Zurich ckreft@ethz.ch

Best regards, Cordelia Kreft (ETH Zurich)

# Assessments of climate change

These questions deal with the consequences of climate change for Swiss agriculture and your farm. There are no right or wrong answers. It’s all about your own personal assessments.

**Q1. Do you think that climate change will have consequences for agriculture in Switzerland?**

Please select the appropriate answer for each item:

| Very negative consequences  1 | 2 | No consequences  3 | 4 | Very positive consequences  5 |
| --- | --- | --- | --- | --- |
|  |  |  |  |  |

**Q2. How did you perceive the frequency of extreme weather events over the past 10 years on your farm?**

|  | Strong increase  1 | 2 | No change  3 | 4 | Strong decrease  5 |
| --- | --- | --- | --- | --- | --- |
| Hail events |  |  |  |  |  |
| Continuous dry phases |  |  |  |  |  |
| Frost in autumn and spring |  |  |  |  |  |
| Heavy rain |  |  |  |  |  |
| Long rainy periods |  |  |  |  |  |
| High temperatures and heat waves |  |  |  |  |  |

**Q3. How do you assess the consequences of climate change for the economic development of your farm?**

| Very negative consequences  1 | 2 | No consequences  3 | 4 | Very positive consequences  5 |
| --- | --- | --- | --- | --- |
|  |  |  |  |  |

**Q4. How would you rate the following statements regarding your role as a farmer in climate change mitigation?**

*Please choose your answer on the scale from 1 ("strongly disagree") to 5 ("fully agree").*

|  | I do not agree at all  1 | 2 | 3 | 4 | I fully agree  5 |
| --- | --- | --- | --- | --- | --- |
| I can do something about climate change on my farm by reducing greenhouse gases. |  |  |  |  |  |
| My behavior as a farmer influences climate change. |  |  |  |  |  |
| How successfully I can reduce greenhouse gases on the farm depends mainly on my skills as a farmer. |  |  |  |  |  |
| I am confident that I can reduce greenhouse gases and at the same time produce successfully. |  |  |  |  |  |
| Climate change is a problem I can not change. |  |  |  |  |  |

# Agricultural climate change mitigation

In this part of the survey, we would like to find out which climate change mitigation measures you implement on your farm and how you assess the effectiveness of the single measures.

Again, there is no right or wrong, it is all about your very personal assessment.

**Q5: Which of the following measures do you currently implement on your farm and do you consider the measures effective for climate change mitigation?**

*Please indicate the appropriate answers for each measure.*

|  | Do you currently implement the measure? | | | How effective do you think the measure is for climate change mitigation? | | | | | |
| --- | --- | --- | --- | --- | --- | --- | --- | --- | --- |
|  | Yes | No | Not relevant for my type of farm. | Not effective at all.  1 | 2 | 3 | 4 | Very effective  5 | I don’t know |
| I substitute some of the (imported) concentrates for my animals with native grain legumes (e.g., peas, lupines, field beans, European soya). |  |  |  |  |  |  |  |  |  |
| I reduce the concentrate content to a maximum of 10 percent of the ration for my animals. |  |  |  |  |  |  |  |  |  |
| I keep my cows for at least 5 lactation periods. |  |  |  |  |  |  |  |  |  |
| I keep cattle of a dual-purpose breed (for example, original brown cattle). |  |  |  |  |  |  |  |  |  |
| I feed my cattle tannins, flaxseed or similar feed additives to reduce methane emissions from digestion. |  |  |  |  |  |  |  |  |  |
| The manure storage on my farm is covered. |  |  |  |  |  |  |  |  |  |
| I compost the farm manure. |  |  |  |  |  |  |  |  |  |
| I apply the fertilizer close to the ground with a drag hose or a similar technology. |  |  |  |  |  |  |  |  |  |
| I include cover or catch crops in my rotation. |  |  |  |  |  |  |  |  |  |
| I do not use the plough for tillage. |  |  |  |  |  |  |  |  |  |
| I have solar panels for energy production. |  |  |  |  |  |  |  |  |  |
| Manure from my farm is fermented in a biogas plant. |  |  |  |  |  |  |  |  |  |
| When working with the tractor I drive in eco-drive mode (fuel-efficient). |  |  |  |  |  |  |  |  |  |

**Q5a: Are you currently implementing any other measures to reduce greenhouse gases on your farm?**

*Please write this in the text field.*

**Q6: Which of the measures that you do not currently implement could you imagine to adopt in the future, which not?**

|  | I can imagine to adopt this measure on my farm. | I can not imagine to adopt this measure on my farm. |
| --- | --- | --- |
| I substitute some of the (imported) concentrates for my animals with native grain legumes (e.g., peas, lupines, field beans, European soya). |  |  |
| I reduce the concentrate content to a maximum of 10 percent of the ration for my animals. |  |  |
| I keep my cows for at least 5 lactation periods. |  |  |
| I keep cattle of a dual-purpose breed (for example, original brown cattle). |  |  |
| I feed my cattle tannins, flaxseed or similar feed additives to reduce methane emissions from digestion. |  |  |
| The manure storage on my farm is covered. |  |  |
| I compost the farm manure. |  |  |
| I apply the fertilizer close to the ground with a drag hose or a similar technology. |  |  |
| I include cover or catch crops in my rotation. |  |  |
| I do not use the plough for tillage. |  |  |
| I have solar panels for energy production. |  |  |
| Manure from my farm is fermented in a biogas plant. |  |  |
| When working with the tractor I drive in eco-drive mode (fuel-efficient). |  |  |

# Personal values and preferences

The following questions serve to assess your personal values ​​and preferences regarding agriculture and climate change. We also aim to find out how you assess your farm and yourself in agricultural climate change mitigation.

**Q7: What is the highest education you have completed?**

| Agricultural apprenticeship |
| --- |
| Agricultural mastership examination |
| Agri-technician |
| Technical college, university, ETH |
| Other: …………………………………………. |

**Q8: Which production activities could you imagine to introduce on your farm?**

*To answer the question, it does not matter what you currently produce on your farm.*

|  | I would definitely do that  ☺ ☺ ☺  1 | ☺ ☺  2 | ☺ ☹  3 | ☹ ☹  4 | I would definitely not do that  ☹ ☹ ☹  5 |
| --- | --- | --- | --- | --- | --- |
| Dairy farming |  |  |  |  |  |
| Cattle fattening |  |  |  |  |  |
| Pig fattening |  |  |  |  |  |
| Poultry |  |  |  |  |  |
| Arable farming |  |  |  |  |  |
| Specialized crops |  |  |  |  |  |
| Off-farm activity |  |  |  |  |  |

**Q9: Please rank the following goals according to how important they are to you when making decisions on the farm.**

*Put the items on the right-hand side (highest rating above). The elements can be moved with the mouse. Double-click moves an element to the other list.*

| To protect the environment and natural resources. |  |
| --- | --- |
| To reduce greenhouse gases on the farm. |  |
| To achieve the highest possible yield. |  |
| To be acknowledged by other farmers in the region. |  |
| To generate the highest possible income from agriculture. |  |
| To preserve a high biodiversity of animals and plants on my land. |  |

**Q10: How well do the following statements apply to you and your farm?**

*Please choose your answer on the scale from 1 ("does not apply at all") to 5 ("fully applies").*

|  | Does not apply at all  1 | 2 | 3 | 4 | Fully applies  5 |
| --- | --- | --- | --- | --- | --- |
| With the management of my farm, I make a contribution to climate change mitigation. |  |  |  |  |  |
| I regularly achieve high yields. |  |  |  |  |  |
| The biodiversity of animals and plants on the land I cultivate is high. |  |  |  |  |  |
| My soil is healthy and fertile. |  |  |  |  |  |
| My agricultural income allows me and my family a good life. |  |  |  |  |  |
| I feel acknowledged by the farmers in the region. |  |  |  |  |  |

**Q11: How well do the following statements apply to you personally?**

*Please choose your answer on the scale from 1 ("does not apply at all") to 5 ("fully applies").*

|  | Does not apply at all  1 | 2 | 3 | 4 | Fully applies  5 |
| --- | --- | --- | --- | --- | --- |
| I am a pioneer in climate change mitigation and implement appropriate measures, even if they involve economic risks. |  |  |  |  |  |
| I am ready to implement climate change mitigation measures earlier than other farmers in the region. |  |  |  |  |  |
| I am open to climate change mitigation, but I want to think through all aspects first. While doing so, I focus on the experiences of other farmers. |  |  |  |  |  |
| In principle, I only implement climate change mitigation measures if they have already been implemented by others for a while and have proven themselves. |  |  |  |  |  |
| I rely on the tried and tested. Implementing climate change mitigation measures on my farm is economically too risky for me. |  |  |  |  |  |

# Income and satisfaction

This part is about your satisfaction with your current income. The first two questions refer to the purely agricultural income per year (including direct payments, excluding off-farm income). The third and fourth questions relate to your total income per year (agricultural income, self-employment and other off-farm income).

**Q12: How satisfied are you currently with your annual agricultural income (including direct payments, excluding off-farm income)?**

| ☺ ☺ ☺  Very satisfied  1 | ☺ ☺  Satisfied  2 | ☺ ☹  So-so  3 | ☹ ☹  Unsatisfied  4 | ☹ ☹ ☹  Very unsatisfied  5 |
| --- | --- | --- | --- | --- |
|  |  |  |  |  |

**Q13: Below what agricultural income per year would you be no longer satisfied (in CHF per year)?**

| 130 000 | 120 000 | 110 000 | 100 000 | 90 000 | 80 000 | 70 000 | 60 000 | 50 000 | 40 000 | 30 000 | 20 000 | 10 000 |
| --- | --- | --- | --- | --- | --- | --- | --- | --- | --- | --- | --- | --- |
|  |  |  |  |  |  |  |  |  |  |  |  |  |

**Q14: How satisfied are you currently with your total earned income (agricultural income, self-employment and other off-farm income)?**

| ☺ ☺ ☺  Very satisfied  1 | ☺ ☺  Satisfied  2 | ☺ ☹  So-so  3 | ☹ ☹  Unsatisfied  4 | ☹ ☹ ☹  Very unsatisfied  5 |
| --- | --- | --- | --- | --- |
|  |  |  |  |  |

**Q15: Below what total income per year would you be no longer satisfied (in CHF per year)?**

| 160 000 | 150 000 | 140 000 | 130 000 | 120 000 | 110 000 | 100 000 | | 90 000 | | 80 000 | 70 000 | 60 000 | 50 000 | 40 000 |
| --- | --- | --- | --- | --- | --- | --- | --- | --- | --- | --- | --- | --- | --- | --- |
|  |  |  |  |  |  |  |  | |  | |  |  |  |  |

**Q16: What is the share of your purely agricultural income (including direct payments, excluding off-farm income) of your total earned income?**

| 0-25% |
| --- |
| 26-50% |
| 51-75% |
| 76-100% |

# The social network

The following questions will help us to understand the role that farmers' social relationships and networks play in agricultural climate change mitigation (e.g. by sharing knowledge and sharing experience or information).

**Q17: How important is it to you what people around you think about the success of your farm and your farming skills?**

| Very important  1 | 2 | 3 | 4 | Not important at all  5 |
| --- | --- | --- | --- | --- |
|  |  |  |  |  |

**Q18: How well do the following statements apply to you personally?**

*Please choose your answer on the scale from 1 ("does not apply at all") to 5 ("fully applies").*

|  | Does not apply at all  1 | 2 | 3 | 4 | Fully applies  5 |
| --- | --- | --- | --- | --- | --- |
| It is important to me to impress other farmers with my farm. |  |  |  |  |  |
| I feel confirmed if I earn more than other farms. |  |  |  |  |  |
| On my farm, I want to produce more environmentally and climate-friendly than other farmers in my area. |  |  |  |  |  |
| If other farmers in my environment earn more than I do, it bothers me. |  |  |  |  |  |
| If other farmers in my environment implement climate change measures, I want to implement such measures on my farm as well. |  |  |  |  |  |

**Q20: With whom do you regularly discuss general agricultural topics and agricultural climate protection?**

Please enter the names (also abbreviations or nicknames possible) of a maximum of 10 people who come to your mind.

| Person 1 |
| --- |
| Person 2 |
| Person 3 |
| Person 4 |
| Person 5 |
| Person 6 |
| Person 7 |
| Person 8 |
| Person 9 |
| Person 10 |

**Q21: Please indicate how you know the person or how you relate to the person (to be completed per person).**

| Neighbour | Workmate | Friend | Family member | Partner | Club-/association colleague | Extension Service | Other |
| --- | --- | --- | --- | --- | --- | --- | --- |
|  |  |  |  |  |  |  |  |

**Q23: How important are the opinions, attitudes and activities of these people when making decisions on your farm?**

*For example, imagine you are faced with deciding whether or not to implement a new climate change mitigation measure on your farm. How important for your decision is what the named person thinks, says or does on his own behalf?*

| Very important | Important | Not important |
| --- | --- | --- |
|  |  |  |

# Risk preferences

# The last three questions are about how you assess risks. Findings on farmers' risk attitudes help to better understand practical decisions on the farm - for example, in agricultural climate change mitigation.

# To answer these last questions lasts a maximum of 10 minutes and you can win up to 190 CHF.

Please have a look at our short explanatory video or carefully read the text below.

- Imagine you want to implement climate change mitigation on you farm.
- To this end, you can invest in either measure A or measure B.
- Both investments promise a certain return, e.g. through higher efficiency and cost savings. Both investments cost the same amount and the respective return is paid out at the same time.
- Investment A generates a return of CHF 40,000 in 3 out of 10 cases (or 30%) and a return of CHF 10,000 in 7 out of 10 cases (or 70%).
- Investment B generates a return of 68,000 CHF in 1 out of 10 cases (or 10%), but only 5,000 CHF in 9 out of 10 cases (90%).
- In the following tables, the less likely return on investment B increases with each series.
- For each question, ask yourself for which return of B you are willing to take the higher risk and invest in B instead of the safer variant A.
- There are no right or wrong answers – it is all about your personal preferences.

**Your decisions determine how much money you can actually win:**

For each of the following three questions, a row is randomly drawn.

- Based on your decision in exactly this row, investment A or B will be used for this question.
- According to the probabilities of the investment (A or B) your profit will be drawn (in question 3 it may also be a loss).
- The amounts from each question are added together and divided by 10,000.

**You can choose between investment A and investment B in each row. Both have the same costs and the respective return is paid out at the same time. However, A and B differ in their predictability: Investment A is stable across the rows. Investment B is less stable, but the potential return from row to row increases.**

**Q24: Please indicate from which row you choose investment B.**

*You can make a selection by clicking on the row in the table. This corresponds to the first row in which you select B.*

|  |  | |  | |
| --- | --- | --- | --- | --- |
|  | **30%** | **70%** | **10%** | **90%** |
| 1 | 40.000 CHF | 10.000 CHF | 68.000 CHF | 5000 CHF |
| 2 | 40.000 CHF | 10.000 CHF | 75.000 CHF | 5000 CHF |
| 3 | 40.000 CHF | 10.000 CHF | 83.000 CHF | 5000 CHF |
| 4 | 40.000 CHF | 10.000 CHF | 93.000 CHF | 5000 CHF |
| 5 | 40.000 CHF | 10.000 CHF | 106.000 CHF | 5000 CHF |
| 6 | 40.000 CHF | 10.000 CHF | 125.000 CHF | 5000 CHF |
| 7 | 40.000 CHF | 10.000 CHF | 150.000 CHF | 5000 CHF |
| 8 | 40.000 CHF | 10.000 CHF | 185.000 CHF | 5000 CHF |
| 9 | 40.000 CHF | 10.000 CHF | 220.000 CHF | 5000 CHF |
| 10 | 40.000 CHF | 10.000 CHF | 300.000 CHF | 5000 CHF |
| 11 | 40.000 CHF | 10.000 CHF | 400.000 CHF | 5000 CHF |
| 12 | 40.000 CHF | 10.000 CHF | 600.000 CHF | 5000 CHF |
| 13 | 40.000 CHF | 10.000 CHF | 1.000.000 CHF | 5000 CHF |
| 14 | 40.000 CHF | 10.000 CHF | 1.700.000 CHF | 5000 CHF |
| Never |  |  |  |  |

**Q25: Please indicate from which row you choose investment B.**

*You can make a selection by clicking on the row in the table. This corresponds to the first row in which you select B.*

|  |  | |  | |
| --- | --- | --- | --- | --- |
|  | **90%** | **10%** | **70%** | **30%** |
| 1 | 40.000 CHF | 30.000 CHF | 54.000 CHF | 5000 CHF |
| 2 | 40.000 CHF | 30.000 CHF | 56.000 CHF | 5000 CHF |
| 3 | 40.000 CHF | 30.000 CHF | 58.000 CHF | 5000 CHF |
| 4 | 40.000 CHF | 30.000 CHF | 60.000 CHF | 5000 CHF |
| 5 | 40.000 CHF | 30.000 CHF | 62.000 CHF | 5000 CHF |
| 6 | 40.000 CHF | 30.000 CHF | 65.000 CHF | 5000 CHF |
| 7 | 40.000 CHF | 30.000 CHF | 68.000 CHF | 5000 CHF |
| 8 | 40.000 CHF | 30.000 CHF | 72.000 CHF | 5000 CHF |
| 9 | 40.000 CHF | 30.000 CHF | 77.000 CHF | 5000 CHF |
| 10 | 40.000 CHF | 30.000 CHF | 83.000 CHF | 5000 CHF |
| 11 | 40.000 CHF | 30.000 CHF | 90.000 CHF | 5000 CHF |
| 12 | 40.000 CHF | 30.000 CHF | 100.000 CHF | 5000 CHF |
| 13 | 40.000 CHF | 30.000 CHF | 110.000 CHF | 5000 CHF |
| 14 | 40.000 CHF | 30.000 CHF | 130.000 CHF | 5000 CHF |
| Never |  |  |  |  |

**Q26: Please indicate from which row you choose investment B.**

*You can make a selection by clicking on the row in the table. This corresponds to the first row in which you select B.*

***Please note: You can also lose money in this question. Therefore, you will now receive 5 CHF, from which any losses due to this task will be deducted when calculating your total gain. In no case can you lose more than this CHF 5.***

|  |  | |  | |
| --- | --- | --- | --- | --- |
|  | **50%** | **50%** | **50%** | **50%** |
| 1 | 25.000 CHF | - 4000 CHF | 3000 CHF | - 21.000 CHF |
| 2 | 4000 CHF | - 4000 CHF | 3000 CHF | - 21.000 CHF |
| 3 | 1000 CHF | - 4000 CHF | 3000 CHF | - 21.000 CHF |
| 4 | 1000 CHF | - 4000 CHF | 3000 CHF | - 16.000 CHF |
| 5 | 1000 CHF | - 8000 CHF | 3000 CHF | - 16.000 CHF |
| 6 | 1000 CHF | - 8000 CHF | 3000 CHF | - 14.000 CHF |
| 7 | 1000 CHF | - 8000 CHF | 3000 CHF | - 11.000 CHF |
| Never |  |  |  |  |

**Q27: After completing the survey, would you like to receive 10 CHF in return for your participation and profit?**

| Yes | No |
| --- | --- |
|  |  |

**Q28: If so, please provide your account details so that we can transfer you CHF 10 and your profit after the poll has ended.**

| Account holder: |
| --- |
| IBAN: |

**Q29:** **Would you like to receive a summary of the survey results?**

| Yes | No |
| --- | --- |
|  |  |

**Q30: Do you have any final feedback or comments?**

|  |
| --- |

Thank you very much for your participation!

Your details and personal data will be kept strictly confidential and will be used exclusively for scientific purposes.

After the survey, we will determine your gains from the last part of the survey. If you answered "Yes" to the question and provided your account details, we will transfer your winnings to your account.

We will also gladly send you a summary of the survey results, if you have indicated your interest accordingly.

For questions and suggestions, please contact:

Cordelia Kreft

Group for Agricultural Economics and Politics, ETH Zurich

ckreft@ethz.ch

You can now close the browser.

Best regards,

Cordelia Kreft
