# Supplementary material for: Data on farmers’ adoption of climate change mitigation measures, individual characteristics, risk attitudes and social influences in a region of Switzerland
Source: Data Brief. 2020 Mar 10;30:105410. doi: 10.1016/j.dib.2020.105410 (PMC7110304; doi:10.1016/j.dib.2020.105410)
Supplement: Supplementary file 3 [file mmc3.docx]

| **Codebook to Data on farmers’ adoption of climate change mitigation measures, individual characteristics, risk attitudes and social influences in a region of Switzerland: Description of variables and questions** | | |
| --- | --- | --- |
| **Variable-names** | **Question** | **Coding** |
| id | Unique answer ID of participant | ID numbers given to each participant |
| date | Date representing when the survey was submitted by participant | Dates |
| total_agr_land | Data obtained from AGIS database | Farm's total agricultural land (in Ar = 0.01 hectares) |
| workforce | Data obtained from AGIS database | Standard workforce units |
| trees | Data obtained from AGIS database | Number of trees owned by the farm |
| arable_land | Data obtained from AGIS database | Farm's total area of arable land (in Ar = 0.01 hectares) |
| ley | Data obtained from AGIS database | Farm's total area of ley (in Ar = 0.01 hectares) |
| perm_grass | Data obtained from AGIS database | Farm's total area of permanent grass land (in Ar = 0.01 hectares) |
| perm_crops | Data obtained from AGIS database | Farm's total area of permanent crops (in Ar = 0.01 hectares) |
| prot_cult | Data obtained from AGIS database | Farm's total area of protected cultures (in Ar = 0.01 hectares) |
| add_agr_land | Data obtained from AGIS database | Farm's total area of additional agricultural land (in Ar = 0.01 hectares) |
| oth_land | Data obtained from AGIS database | Farm's total are of other land (in Ar = 0.01 hectares) |
| qual_landscape | Data obtained from AGIS database | Farm's total are of land enrolled in payments for landscape quality (in Ar = 0.01 hectares) |
| spec_crops | Data obtained from AGIS database | Farm's total area of specialized crops (in Ar = 0.01 hectares) |
| grapes | Data obtained from AGIS database | Farm's total are of grapes (in Ar = 0.01 hectares) |
| vegetables | Data obtained from AGIS database | Farm's total area of vegetables (in Ar = 0.01 hectares) |
| fruits | Data obtained from AGIS database | Farm's total are of fruits (in Ar = 0.01 hectares) |
| oth_spec_crops | Data obtained from AGIS database | Farm's total are of other specialized crops (in Ar = 0.01 hectares) |
| animal_unit | Data obtained from AGIS database | Total animal units on the farm |
| cattle_animal_unit | Data obtained from AGIS database | Total animal units of cattle on the farm |
| dairy_cows | Data obtained from AGIS database | Total animal units of dairy cows on the farm |
| suckler_cows | Data obtained from AGIS database | Total animal units of suckler cows on the farm |
| hor_shee_goa | Data obtained from AGIS database | Total animal units of horses, sheep or goats on the farm |
| pigs_poultry | Data obtained from AGIS database | Total animal units of pigs and poultry on the farm |
| pigs | Data obtained from AGIS database | Total animal units of pigs on the farm |
| poultry | Data obtained from AGIS database | Total animal units of poultry on the farm |
| eco_proof | Data obtained from AGIS database | 1= Farm produces according to Federal regulations of Proof of Ecological Performance (PEP) 0= Farm does not produce according to Federal regulations of Proof of Ecological Performance (PEP) |
| organic | Data obtained from AGIS database | 1= Farm produces according to Swiss Organic Farming Ordinance  0= Farm does not produce according to Swiss Organic Farming Ordinance |
| farmtype | Data obtained from AGIS database (main production based on relative area cultivated and livestock units, respectively) | Arable farming = Farm mainly produces arable crops Livestock = Farm mainly produces livestock Specialized Crops = Farm mainly produces specialized crops  Others = Farm mainly produces other products |
| region | Data obtained from AGIS database | Kohlfirst/Rhein Flaachtal Thurtal Stammertal Andere Gemeinde |
| age | Data obtained from AGIS database | Numbers |
| educ | What is the highest education you have completed? | 1= Agricultural apprenticeship 2= Agricultural mastership examination 3= Agri-technician 4= Technical college, university, Swiss Federal Institute of Technology 5= Other |
| cons_general | Do you think that climate change will have consequences for agriculture in Switzerland? | 1 = very positive consequences, 2, 3 = no consequences, 4, 5= very negative consequences |
| percep_hail | How did you perceive the frequency of extreme weather events over the past 10 years on your farm? [Hail] | 1= no change, 2 = increase or decrease, 3 = strong increase or strong decrease |
| percep_drought | How often have you perceived extreme weather events over the past 10 years? [Drought] | 1= no change, 2 = increase or decrease, 3 = strong increase or strong decrease |
| percep_frost | How often have you perceived extreme weather events over the past 10 years? [Frost in spring or autumn] | 1= no change, 2 = increase or decrease, 3 = strong increase or strong decrease |
| percep_heavyrain | How often have you perceived extreme weather events over the past 10 years? [Heavy rain] | 1= no change, 2 = increase or decrease, 3 = strong increase or strong decrease |
| percep_longrain | How often have you perceived extreme weather events over the past 10 years? [Prolonged rain periods] | 1= no change, 2 = increase or decrease, 3 = strong increase or strong decrease |
| percep_heat | How often have you perceived extreme weather events over the past 10 years? [Heat waves] | 1= no change, 2 = increase or decrease, 3 = strong increase or strong decrease |
| cons_farm | How do you assess the consequences of climate change for the economic development of your farm? | 1 = very positive consequences, 2, 3 = no consequences, 4, 5= very negative consequences |
| self_reduc | I can do something about climate change on my farm by reducing greenhouse gases. | 1= I fully disagree, 2, 3, 4, 5= I fully agree |
| self_act | My behavior as a farmer influences climate change. | 1= I fully disagree, 2, 3, 4, 5= I fully agree |
| self_cap | How successfully I can reduce greenhouse gases on the farm depends mainly on my skills as a farmer. | 1= I fully disagree, 2, 3, 4, 5= I fully agree |
| self_conf | I am confident that I can reduce greenhouse gases and at the same time produce successfully. | 1= I fully disagree, 2, 3, 4, 5= I fully agree |
| self_not | Climate change is a problem I can not change. | 1= I fully agree, 2, 3, 4, 5= I fully disagree |
| legum | Do you currently implement this measure [Legumes] | 1=yes; 0= no, NA= not relevant |
| legum_eff | How effective do you think is this measure? [Legumes] | 1= not effective at all, 2, 3, 4, 5 = very effective, NA= don't know |
| conc | Do you currently implement this measure [Reduction concentrates] | 1=yes; 0= no, NA= not relevant |
| conc_eff | How effective do you think is this measure? [Reduction concentrates] | 1= not effective at all, 2, 3, 4, 5 = very effective, NA= don't know |
| add | Do you currently implement this measure [Additives] | 1=yes; 0= no, NA= not relevant |
| add_eff | How effective do you think is this measure? [Additives] | 1= not effective at all, 2, 3, 4, 5 = very effective, NA= don't know |
| lact | Do you currently implement this measure [Lactation] | 1=yes; 0= no, NA= not relevant |
| lact_eff | How effective do you think is this measure? [Lactation] | 1= not effective at all, 2, 3, 4, 5 = very effective, NA= don't know |
| breed | Do you currently implement this measure [Breed] | 1=yes; 0= no, NA= not relevant |
| breed_eff | How effective do you think is this measure? [Breed] | 1= not effective at all, 2, 3, 4, 5 = very effective, NA= don't know |
| covman | Do you currently implement this measure [Cover manure] | 1=yes; 0= no, NA= not relevant |
| covman_eff | How effective do you think is this measure? [Cover manure] | 1= not effective at all, 2, 3, 4, 5 = very effective, NA= don't know |
| comp | Do you currently implement this measure [Compost manure] | 1=yes; 0= no, NA= not relevant |
| comp_eff | How effective do you think is this measure? [Compost manure] | 1= not effective at all, 2, 3, 4, 5 = very effective, NA= don't know |
| drag | Do you currently implement this measure [Drag hose] | 1=yes; 0= no, NA= not relevant |
| drag_eff | How effective do you think is this measure? [Drag hose] | 1= not effective at all, 2, 3, 4, 5 = very effective, NA= don't know |
| cov | Do you currently implement this measure [Cover crop] | 1=yes; 0= no, NA= not relevant |
| cov_eff | How effective do you think is this measure? [Cover crop] | 1= not effective at all, 2, 3, 4, 5 = very effective, NA= don't know |
| plough | Do you currently implement this measure [Ploughless] | 1=yes; 0= no, NA= not relevant |
| plough_eff | How effective do you think is this measure? [Ploughless] | 1= not effective at all, 2, 3, 4, 5 = very effective, NA= don't know |
| solar | Do you currently implement this measure [Solarpanels] | 1=yes; 0= no, NA= not relevant |
| solar_eff | How effective do you think is this measure? [Solarpanels] | 1= not effective at all, 2, 3, 4, 5 = very effective, NA= don't know |
| biog | Do you currently implement this measure [Biogas] | 1=yes; 0= no, NA= not relevant |
| biog_eff | How effective do you think is this measure? [Biogas] | 1= not effective at all, 2, 3, 4, 5 = very effective, NA= don't know |
| ecodr | Do you currently implement this measure [Ecodrive] | 1=yes; 0= no, NA= not relevant |
| ecodr_eff | How effective do you think is this measure? [Ecodrive] | 1= not effective at all, 2, 3, 4, 5 = very effective, NA= don't know |
| legum_imag | Which of the measures that you do not currently implement could you imagine to adopt in the future, which not? [Legumes] | 1= I can imagine to implement it, 0 = I cannot imagine to implement it, NA = not relevant or already implemented |
| conc_imag | Which of the measures that you do not currently implement could you imagine to adopt in the future, which not? [Concentrates] | 1= I can imagine to implement it, 0 = I cannot imagine to implement it, NA = not relevant or already implemented |
| add_imag | Which of the measures that you do not currently implement could you imagine to adopt in the future, which not? [Additives] | 1= I can imagine to implement it, 0 = I cannot imagine to implement it, NA = not relevant or already implemented |
| lact_imag | Which of the measures that you do not currently implement could you imagine to adopt in the future, which not? [Lactation] | 1= I can imagine to implement it, 0 = I cannot imagine to implement it, NA = not relevant or already implemented |
| breed_imag | Which of the measures that you do not currently implement could you imagine to adopt in the future, which not? [Breed] | 1= I can imagine to implement it, 0 = I cannot imagine to implement it, NA = not relevant or already implemented |
| covman_imag | Which of the measures that you do not currently implement could you imagine to adopt in the future, which not? [Cover manure] | 1= I can imagine to implement it, 0 = I cannot imagine to implement it, NA = not relevant or already implemented |
| comp_imag | Which of the measures that you do not currently implement could you imagine to adopt in the future, which not? [Compost manure] | 1= I can imagine to implement it, 0 = I cannot imagine to implement it, NA = not relevant or already implemented |
| drag_imag | Which of the measures that you do not currently implement could you imagine to adopt in the future, which not? [Drag hose] | 1= I can imagine to implement it, 0 = I cannot imagine to implement it, NA = not relevant or already implemented |
| cov_imag | Which of the measures that you do not currently implement could you imagine to adopt in the future, which not? [Cover crop] | 1= I can imagine to implement it, 0 = I cannot imagine to implement it, NA = not relevant or already implemented |
| plough_imag | Which of the measures that you do not currently implement could you imagine to adopt in the future, which not? [Ploughless] | 1= I can imagine to implement it, 0 = I cannot imagine to implement it, NA = not relevant or already implemented |
| solar_imag | Which of the measures that you do not currently implement could you imagine to adopt in the future, which not? [Solarpanels] | 1= I can imagine to implement it, 0 = I cannot imagine to implement it, NA = not relevant or already implemented |
| biog_imag | Which of the measures that you do not currently implement could you imagine to adopt in the future, which not? [Biogas] | 1= I can imagine to implement it, 0 = I cannot imagine to implement it, NA = not relevant or already implemented |
| ecodr_imag | Which of the measures that you do not currently implement could you imagine to adopt in the future, which not? [Ecodrive] | 1= I can imagine to implement it, 0 = I cannot imagine to implement it, NA = not relevant or already implemented |
| activ_dairy | Which activities can you prinicipally imagine for your company and which not? [Dairy] | 1= Would definitely do, 2, 3, 4, 5= Would definitely not do that |
| activ_cattle | Which activities can you prinicipally imagine for your company and which not? [Cattle] | 1= Would definitely do, 2, 3, 4, 5= Would definitely not do that |
| activ_pig | Which activities can you prinicipally imagine for your company and which not? [Pigs] | 1= Would definitely do, 2, 3, 4, 5= Would definitely not do that |
| activ_poultry | Which activities can you prinicipally imagine for your company and which not? [Poultry] | 1= Would definitely do, 2, 3, 4, 5= Would definitely not do that |
| activ_arab | Which activities can you prinicipally imagine for your company and which not? [Arable farming] | 1= Would definitely do, 2, 3, 4, 5= Would definitely not do that |
| activ_special | Which activities can you prinicipally imagine for your company and which not? [Special crops] | 1= Would definitely do, 2, 3, 4, 5= Would definitely not do that |
| activ_nonag | Which activities can you prinicipally imagine for your company and which not?[Non-agricultural] | 1= Would definitely do, 2, 3, 4, 5= Would definitely not do that |
| attain_ghg | How well do the statements apply to you and your farm? [Climate protection] | 1=does not apply at all , 2,3,4,5= fully applies |
| attain_yield | How well do the statements apply to you and your farm? [Yield] | 1=does not apply at all , 2,3,4,5= fully applies |
| attain_biodiv | How well do the statements apply to you and your farm? [Biodiversity] | 1=does not apply at all , 2,3,4,5= fully applies |
| attain_soil | How well do the statements apply to you and your farm? [Soil fertility] | 1=does not apply at all , 2,3,4,5= fully applies |
| attain_incom | How well do the statements apply to you and your farm? [Income] | 1=does not apply at all , 2,3,4,5= fully applies |
| attain_acknow | How well do the statements apply to you and your farm? [Social acknowledgement] | 1=does not apply at all , 2,3,4,5= fully applies |
| innov_pion | How well do the statements apply to you and your farm? [Pinoneer] | 1=does not apply at all , 2,3,4,5= fully applies |
| innov_early | How well do the statements apply to you and your farm? [Early adopter] | 1=does not apply at all , 2,3,4,5= fully applies |
| innov_thorou | How well do the statements apply to you and your farm? [Thoroughly thought through] | 1=does not apply at all , 2,3,4,5= fully applies |
| innov_others | How well do the statements apply to you and your farm? [After others] | 1=does not apply at all , 2,3,4,5= fully applies |
| innov_trad | How well do the statements apply to you and your farm? [Traditional] | 1=fully applies , 2,3,4,5= does not apply at all |
| satisf_ldw | How satisfied are you currently with your annual agricultural income (including direct payments, excluding off-farm income)? | 1 =very unhappy, 2, 3, 4, 5=very happy |
| thresh_ldw | Below what agricultural income per year would you be no longer satisfied (in CHF per year)? | 13=130000, 12=120000, 11=110000, 10=100000, 9=90000, 8=80000, 7=70000, 6=60000, 5=50000, 4=40000, 3=30000, 2=20000, 1=10000 |
| sartisf_total | How satisfied are you currently with your total earned income (agricultural income, self-employment and other off-farm income)? | 1 =very unhappy, 2, 3, 4, 5=very happy |
| thresh_total | Below what total income per year would you be no longer satisfied (in CHF per year)? | 13=160000, 12=150000, 11=140000, 10=130000, 9=120000, 8=110000,7=100000, 6=90000, 5=80000, 4=70000, 3=60000, 2=50000,1= 40000 |
| share | What is the share of your purely agricultural income (including direct payments, excluding off-farm income) of your total earned income? | 1=0-25%, 2=25-50%, 3=51-75%, 4=76-100% |
| others_opinions | How important is it to you what people around you think about the success of your farm and your farming skills? | 1=not important at all, 2,3,4,5=very important |
| soc_impr | How well do the statements apply to you and your farm? [Impression] | 1=does not apply at all , 2,3,4,5= fully applies |
| soc_inc | How well do the statements apply to you and your farm? [higher income] | 1=does not apply at all , 2,3,4,5= fully applies |
| soc_env | How well do the statements apply to you and your farm? [more enviromental] | 1=does not apply at all , 2,3,4,5= fully applies |
| soc_compinc | How well do the statements apply to you and your farm? [comparison income] | 1=does not apply at all , 2,3,4,5= fully applies |
| soc_compenv | How well do the statements apply to you and your farm? [comparison environmental] | 1=does not apply at all , 2,3,4,5= fully applies |
| net_name1_neigh | Please indicate from where you know the person or how you are related to this person [Neighbor] | 1=yes; NA=no answer |
| net_name1_work | Please indicate from where you know the person or how you are related to this person [Workmate] | 1=yes; NA=no answer |
| net_name1_frien | Please indicate from where you know the person or how you are related to this person [Friend] | 1=yes; NA=no answer |
| net_name1_fam | Please indicate from where you know the person or how you are related to this person [Familymember] | 1=yes; NA=no answer |
| net_name1_part | Please indicate from where you know the person or how you are related to this person [Partner] | 1=yes; NA=no answer |
| net_name1_club | Please indicate from where you know the person or how you are related to this person [Club Colleague] | 1=yes; NA=no answer |
| net_name1_vet | Please indicate from where you know the person or how you are related to this person [Veterinary] | 1=yes; NA=no answer |
| net_name1_ext | Please indicate from where you know the person or how you are related to this person [Extension service] | 1=yes; NA=no answer |
| net_name1_oth | Please indicate from where you know the person or how you are related to this person [Other] | 1=yes; NA=no answer |
| net_name2_neigh | Please indicate from where you know the person or how you are related to this person [Neighbor] | 1=yes; NA=no answer |
| net_name2_work | Please indicate from where you know the person or how you are related to this person [Workmate] | 1=yes; NA=no answer |
| net_name2_frien | Please indicate from where you know the person or how you are related to this person [Friend] | 1=yes; NA=no answer |
| net_name2_fam | Please indicate from where you know the person or how you are related to this person [Familymember] | 1=yes; NA=no answer |
| net_name2_part | Please indicate from where you know the person or how you are related to this person [Partner] | 1=yes; NA=no answer |
| net_name2_club | Please indicate from where you know the person or how you are related to this person [Club Colleague] | 1=yes; NA=no answer |
| net_name2_vet | Please indicate from where you know the person or how you are related to this person [Veterinary] | 1=yes; NA=no answer |
| net_name2_ext | Please indicate from where you know the person or how you are related to this person [Extension service] | 1=yes; NA=no answer |
| net_name2_oth | Please indicate from where you know the person or how you are related to this person [Other] | 1=yes; NA=no answer |
| net_name3_neigh | Please indicate from where you know the person or how you are related to this person [Neighbor] | 1=yes; NA=no answer |
| net_name3_work | Please indicate from where you know the person or how you are related to this person [Workmate] | 1=yes; NA=no answer |
| net_name3_frien | Please indicate from where you know the person or how you are related to this person [Friend] | 1=yes; NA=no answer |
| net_name3_fam | Please indicate from where you know the person or how you are related to this person [Familymember] | 1=yes; NA=no answer |
| net_name3_part | Please indicate from where you know the person or how you are related to this person [Partner] | 1=yes; NA=no answer |
| net_name3_club | Please indicate from where you know the person or how you are related to this person [Club Colleague] | 1=yes; NA=no answer |
| net_name3_vet | Please indicate from where you know the person or how you are related to this person [Veterinary] | 1=yes; NA=no answer |
| net_name3_ext | Please indicate from where you know the person or how you are related to this person [Extension service] | 1=yes; NA=no answer |
| net_name3_oth | Please indicate from where you know the person or how you are related to this person [Other] | 1=yes; NA=no answer |
| net_name4_neigh | Please indicate from where you know the person or how you are related to this person [Neighbor] | 1=yes; NA=no answer |
| net_name4_work | Please indicate from where you know the person or how you are related to this person [Workmate] | 1=yes; NA=no answer |
| net_name4_frien | Please indicate from where you know the person or how you are related to this person [Friend] | 1=yes; NA=no answer |
| net_name4_fam | Please indicate from where you know the person or how you are related to this person [Familymember] | 1=yes; NA=no answer |
| net_name4_part | Please indicate from where you know the person or how you are related to this person [Partner] | 1=yes; NA=no answer |
| net_name4_club | Please indicate from where you know the person or how you are related to this person [Club Colleague] | 1=yes; NA=no answer |
| net_name4_vet | Please indicate from where you know the person or how you are related to this person [Veterinary] | 1=yes; NA=no answer |
| net_name4_ext | Please indicate from where you know the person or how you are related to this person [Extension service] | 1=yes; NA=no answer |
| net_name4_oth | Please indicate from where you know the person or how you are related to this person [Other] | 1=yes; NA=no answer |
| net_name5_neigh | Please indicate from where you know the person or how you are related to this person [Neighbor] | 1=yes; NA=no answer |
| net_name5_work | Please indicate from where you know the person or how you are related to this person [Workmate] | 1=yes; NA=no answer |
| net_name5_frien | Please indicate from where you know the person or how you are related to this person [Friend] | 1=yes; NA=no answer |
| net_name5_fam | Please indicate from where you know the person or how you are related to this person [Familymember] | 1=yes; NA=no answer |
| net_name5_part | Please indicate from where you know the person or how you are related to this person [Partner] | 1=yes; NA=no answer |
| net_name5_club | Please indicate from where you know the person or how you are related to this person [Club Colleague] | 1=yes; NA=no answer |
| net_name5_vet | Please indicate from where you know the person or how you are related to this person [Veterinary] | 1=yes; NA=no answer |
| net_name5_ext | Please indicate from where you know the person or how you are related to this person [Extension service] | 1=yes; NA=no answer |
| net_name5_oth | Please indicate from where you know the person or how you are related to this person [Other] | 1=yes; NA=no answer |
| net_name6_neigh | Please indicate from where you know the person or how you are related to this person [Neighbor] | 1=yes; NA=no answer |
| net_name6_work | Please indicate from where you know the person or how you are related to this person [Workmate] | 1=yes; NA=no answer |
| net_name6_frien | Please indicate from where you know the person or how you are related to this person [Friend] | 1=yes; NA=no answer |
| net_name6_fam | Please indicate from where you know the person or how you are related to this person [Familymember] | 1=yes; NA=no answer |
| net_name6_part | Please indicate from where you know the person or how you are related to this person [Partner] | 1=yes; NA=no answer |
| net_name6_club | Please indicate from where you know the person or how you are related to this person [Club Colleague] | 1=yes; NA=no answer |
| net_name6_vet | Please indicate from where you know the person or how you are related to this person [Veterinary] | 1=yes; NA=no answer |
| net_name6_ext | Please indicate from where you know the person or how you are related to this person [Extension service] | 1=yes; NA=no answer |
| net_name6_oth | Please indicate from where you know the person or how you are related to this person [Other] | 1=yes; NA=no answer |
| net_name7_neigh | Please indicate from where you know the person or how you are related to this person [Neighbor] | 1=yes; NA=no answer |
| net_name7_work | Please indicate from where you know the person or how you are related to this person [Workmate] | 1=yes; NA=no answer |
| net_name7_frien | Please indicate from where you know the person or how you are related to this person [Friend] | 1=yes; NA=no answer |
| net_name7_fam | Please indicate from where you know the person or how you are related to this person [Familymember] | 1=yes; NA=no answer |
| net_name7_part | Please indicate from where you know the person or how you are related to this person [Partner] | 1=yes; NA=no answer |
| net_name7_club | Please indicate from where you know the person or how you are related to this person [Club Colleague] | 1=yes; NA=no answer |
| net_name7_vet | Please indicate from where you know the person or how you are related to this person [Veterinary] | 1=yes; NA=no answer |
| net_name7_ext | Please indicate from where you know the person or how you are related to this person [Extension service] | 1=yes; NA=no answer |
| net_name7_oth | Please indicate from where you know the person or how you are related to this person [Other] | 1=yes; NA=no answer |
| net_name8_neigh | Please indicate from where you know the person or how you are related to this person [Neighbor] | 1=yes; NA=no answer |
| net_name8_work | Please indicate from where you know the person or how you are related to this person [Workmate] | 1=yes; NA=no answer |
| net_name8_frien | Please indicate from where you know the person or how you are related to this person [Friend] | 1=yes; NA=no answer |
| net_name8_fam | Please indicate from where you know the person or how you are related to this person [Familymember] | 1=yes; NA=no answer |
| net_name8_part | Please indicate from where you know the person or how you are related to this person [Partner] | 1=yes; NA=no answer |
| net_name8_club | Please indicate from where you know the person or how you are related to this person [Club Colleague] | 1=yes; NA=no answer |
| net_name8_vet | Please indicate from where you know the person or how you are related to this person [Veterinary] | 1=yes; NA=no answer |
| net_name8_ext | Please indicate from where you know the person or how you are related to this person [Extension service] | 1=yes; NA=no answer |
| net_name8_oth | Please indicate from where you know the person or how you are related to this person [Other] | 1=yes; NA=no answer |
| net_name9_neigh | Please indicate from where you know the person or how you are related to this person [Neighbor] | 1=yes; NA=no answer |
| net_name9_work | Please indicate from where you know the person or how you are related to this person [Workmate] | 1=yes; NA=no answer |
| net_name9_frien | Please indicate from where you know the person or how you are related to this person [Friend] | 1=yes; NA=no answer |
| net_name9_fam | Please indicate from where you know the person or how you are related to this person [Familymember] | 1=yes; NA=no answer |
| net_name9_part | Please indicate from where you know the person or how you are related to this person [Partner] | 1=yes; NA=no answer |
| net_name9_club | Please indicate from where you know the person or how you are related to this person [Club Colleague] | 1=yes; NA=no answer |
| net_name9_vet | Please indicate from where you know the person or how you are related to this person [Veterinary] | 1=yes; NA=no answer |
| net_name9_ext | Please indicate from where you know the person or how you are related to this person [Extension service] | 1=yes; NA=no answer |
| net_name9_oth | Please indicate from where you know the person or how you are related to this person [Other] | 1=yes; NA=no answer |
| net_name10_neigh | Please indicate from where you know the person or how you are related to this person [Neighbor] | 1=yes; NA=no answer |
| net_name10_work | Please indicate from where you know the person or how you are related to this person [Workmate] | 1=yes; NA=no answer |
| net_name10_frien | Please indicate from where you know the person or how you are related to this person [Friend] | 1=yes; NA=no answer |
| net_name10_fam | Please indicate from where you know the person or how you are related to this person [Familymember] | 1=yes; NA=no answer |
| net_name10_part | Please indicate from where you know the person or how you are related to this person [Partner] | 1=yes; NA=no answer |
| net_name10_club | Please indicate from where you know the person or how you are related to this person [Club Colleague] | 1=yes; NA=no answer |
| net_name10_vet | Please indicate from where you know the person or how you are related to this person [Veterinary] | 1=yes; NA=no answer |
| net_name10_ext | Please indicate from where you know the person or how you are related to this person [Extension service] | 1=yes; NA=no answer |
| net_name10_oth | Please indicate from where you know the person or how you are related to this person [Other] | 1=yes; NA=no answer |
| net_name1_imp | Please indicate how important the person is for your decision-making on the farm. | 1=very important, 2=important, 3= not important |
| net_name2_imp | Please indicate how important the person is for your decision-making on the farm. | 1=very important, 2=important, 3= not important |
| net_name3_imp | Please indicate how important the person is for your decision-making on the farm. | 1=very important, 2=important, 3= not important |
| net_name4_imp | Please indicate how important the person is for your decision-making on the farm. | 1=very important, 2=important, 3= not important |
| net_name5_imp | Please indicate how important the person is for your decision-making on the farm. | 1=very important, 2=important, 3= not important |
| net_name6_imp | Please indicate how important the person is for your decision-making on the farm. | 1=very important, 2=important, 3= not important |
| net_name7_imp | Please indicate how important the person is for your decision-making on the farm. | 1=very important, 2=important, 3= not important |
| net_name8_imp | Please indicate how important the person is for your decision-making on the farm. | 1=very important, 2=important, 3= not important |
| net_name9_imp | Please indicate how important the person is for your decision-making on the farm. | 1=very important, 2=important, 3= not important |
| net_name10_imp | Please indicate how important the person is for your decision-making on the farm. | 1=very important, 2=important, 3= not important |
| lott_1 | Please indicate in which row you would switch from option A to option B. | 1,2,3,4,5,6,7,8,9,10, 11, 12, 13, 14, never |
| lott_2 | Please indicate in which row you would switch from option A to option B. | 1,2,3,4,5,6,7,8,9,10, 11, 12, 13, 14, never |
| lott_3 | Please indicate in which row you would switch from option A to option B. | 1,2,3,4,5,6,7,never |
| agroconcept | Obtained from data on AgroCO2ncept project | 0=Not participating, 1 = participating |
| network | Sum of all contact persons named | Numbers |
| GHG_goal | Please rank these goals regarding their importance for your decisions on the farm. [Reduction of GHG emissions] | 6 = highest importance, 5, 4, 3, 2, 1= lowest importance |
| env_goal | Please rank these goals regarding their importance for your decisions on the farm. [Protection of environment] | 7 = highest importance, 5, 4, 3, 2, 1= lowest importance |
| biodiv_goal | Please rank these goals regarding their importance for your decisions on the farm. [Preservation of biodiversity] | 8 = highest importance, 5, 4, 3, 2, 1= lowest importance |
| acknow_goal | Please rank these goals regarding their importance for your decisions on the farm. [Social acknowledgement] | 9 = highest importance, 5, 4, 3, 2, 1= lowest importance |
| yield_goal | Please rank these goals regarding their importance for your decisions on the farm. [High yield] | 10 = highest importance, 5, 4, 3, 2, 1= lowest importance |
| income_goal | Please rank these goals regarding their importance for your decisions on the farm. [High income] | 11 = highest importance, 5, 4, 3, 2, 1= lowest importance |
| info_gew | After completing the survey, would you like to receive 10 CHF in return for your participation and profit? | 1=yes; 0= no |
| info_resul | Would you like to receive a summary of the survey results? | 1=yes; 0= no |
| interviewtime | Data representing how long the participant took to answer the whole survey | Seconds |
